# Mapping heavy mineral deposits on the coast of the state of Rio Grande do Sul (Brazil) using orbital and proximal remote sensing

**DOI:** 10.1371/journal.pone.0309043

**Published:** 2024-09-06

**Authors:** Gabriel Prates Hallal, Jean Marcel de Almeida Espinoza, Bijeesh Kozhikkodan Veettil, Carla Cristine Porcher, Maurício Oliveira Righi da Silva, Silvia Beatriz Alves Rolim

**Affiliations:** 1 State Research Center for Remote Sensing and Meteorology (CEPSRM), Federal University of Rio Grande do Sul (UFRGS), Porto Alegre, Brazil; 2 Laboratório de Física Experimental, Instituto Federal de Educação Ciência e Tecnologia de Santa Catarina, Garopaba, Brazil; 3 Laboratory of Ecology and Environmental Management, Science and Technology Advanced Institute, Van Lang University, Ho Chi Minh City, Vietnam; 4 Faculty of Applied Technology, School of Technology, Van Lang University, Ho Chi Minh City, Vietnam; 5 Centro de Estudos em Petrologia e Geoquímica, Instituto de Geologia, Universidade Federal do Rio Grande do Sul, Porto Alegre, Brazil; Birbal Sahni Institute of Palaeosciences: Birbal Sahni Institute of Palaeobotany, INDIA

## Abstract

Heavy mineral deposits occur in several coastal areas of the world, formed over a long period due to variations in mean sea level, wave action, and winds. These are the main sources of ilmenite (FeTiO_3_), which in turn is the source of more than 80% of the TiO_2_ produced and applied in various industries, most recently in nanotechnology. The present study mapped heavy mineral deposits on the coast of Rio Grande do Sul in southern Brazil using integrated proximal and orbital thermal infrared (TIR) remote sensing techniques. Mineral groups, such as oxides and silicates, have spectral features in the TIR wavelengths. Using laboratory spectroscopy at TIR using Nicolet 6700 Thermo Scientific Spectrometer, we measured the spectral signature of the local sample of heavy minerals (between 8 and 14 μm) and identified a diagnostic spectral feature at 10.75 μm. The signature was resampled to be compatible with the Advanced Spaceborne Thermal Emission Radiometer (ASTER) sensor bandwidth values and used as a reference endmember for the Spectral Angle Mapper (SAM) and Linear Spectral Unmixing (LSU) digital image classification algorithms. Thus, we identified the presence of the reference endmember (heavy minerals) in the pixels of the ASTER scene. In pixels classified by SAM as the presence of heavy minerals, LSU was applied to estimate the surface concentration within the pixel. The results showed a concentration of up to 20% of heavy minerals, with the highest concentration on the beach and dune fields. Opaque minerals such as ilmenite do not have spectral reflectance features in visible, near-infrared, and short-wave infrared, which makes their identification by remote sensing difficult. The present study showed that the integration of proximal and orbital as well as hyperspectral and multispectral thermal data can be considered as an alternative for detecting and mapping heavy minerals in coastal areas.

## 1. Introduction

Titanium is a heavy metal having high economic and industrial importance, with its main application (more than 90%) in the form of titanium dioxide (TiO_2_) [1, 2]. Heavy mineral deposits rich in titanium, such as ilmenite and rutile, were formed in many coastal areas of the world such as the southeastern coast of Bangladesh [3], associated with specific conditions of geomorphological evolution [3, 4]. More than 80% of the TiO_2_ produced worldwide is obtained from the processing of ilmenite (FeTiO_3_) and, due to its chemical properties, it has applications in multiple industries such as paints, human prosthetics, rocket and aircraft fuselages, cosmetics, treatment sewage as a photocatalyst, coatings as a corrosion prevention agent [5, 6]. More recently, its application in electronic devices and the production of energy through oxygen combustion has led to an increase in the number of patents for the use of TiO_2_ [2, 7].

The highest industrial production of TiO_2_ is in China (more than 50%), followed by the United States and Australia, while the largest mining activities are also found in China (35%), followed by South Africa (13%), Mozambique (12%), Canada (8%) and Australia (6%) [2, 8]. In Brazil, heavy minerals are currently being explored only at the Guajú mine, in the state of Paraíba, although efforts are being made toward independent national production based on the forecast of TiO_2_ consumption growth in the country [9–11].

The coast of Rio Grande do Sul state in Brazil is known for a high concentration of heavy minerals [12], where mining, exploration, and research requirements must be acquired from the National Department of Mineral Production (DNPM) by the company Rio Grande Mineração S/A. However, the exploration awaits environmental licenses from competent bodies [10, 11]. In this context, the Retiro project foresees the exploration of eleven of these requirements in a total area of 49 km² on the central coast of the state of Rio Grande do Sul [11].

Advances in imaging spectroscopy have driven the development of digital image processing algorithms for mapping heavy minerals on the Earth’s surface. Among these, we can highlight the Spectral Angle Mapper (SAM) algorithms [13, 14] and Linear Spectral Unmixing (LSU) [15, 16]. SAM calculates the similarity between two spectra and expresses this similarity in terms of average angle generating a pixel-by-pixel classification [17, 18]. SAM has the advantages of simplicity of use, processing speed, and availability in commercial software packages [19, 20]. However, as a disadvantage, it has the limitation of disregarding the spectral mixing within the pixel [21]. LSU is a sub-pixel classifier capable of estimating the abundance of a reference endmember within the pixel, assuming that the pixel’s spectral response is a linear combination of each contained endmember [22].

Given the economic relevance of heavy minerals and the potential of remote sensing techniques to support geological mapping, this work aims to integrate proximal (laboratory data) and orbital (ASTER data) spectroscopy techniques, using the TIR spectral range as an innovation in prospecting for titanium-rich mineral deposits, using the coastal plain of the state of Rio Grande do Sul in Brazil as the study area. The present study utilized both SAM and LSU algorithms for mapping heavy minerals on the coast of Southern Brazil.

## 2. Study area

**Fig 1:**
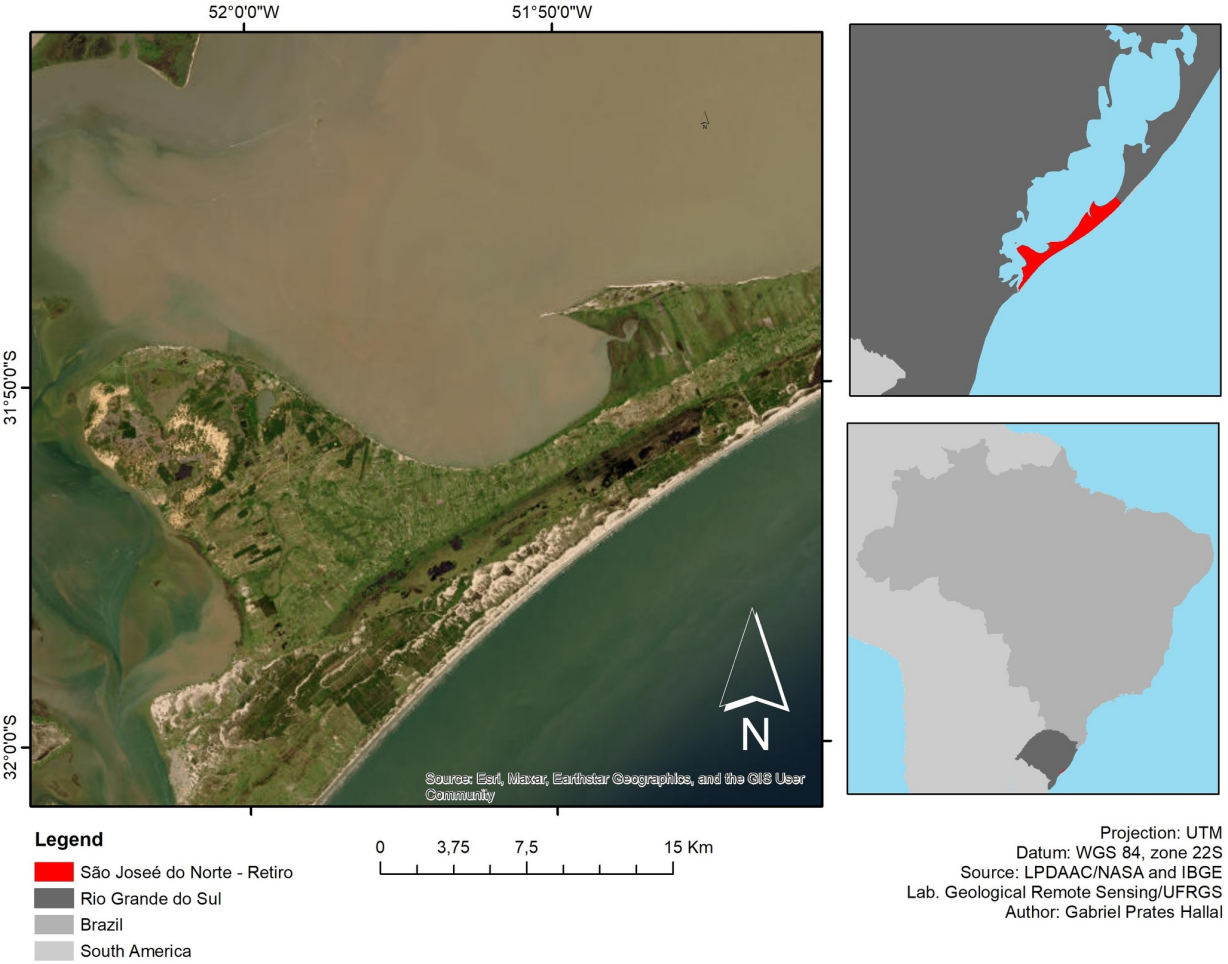
Geographical location of the study area in Southern Brazil.

The municipality of São José do Norte (SJN) is located on the coastal plain of the state of Rio Grande do Sul (Fig 1), on the west bank of the access channel to the Patos Lagoon estuary, and has an estimated population of approximately 28000 inhabitants [23]. Economic activity is based on agriculture, livestock, and fishing, in addition to the cultivation of *Pinus Elliottii* for timber and resin extraction. SJN has a close relationship with the municipality of Rio Grande located on the east bank of the canal due to the activities of the port. The Federal Highway BR-101 ends at SJN, the second longest highway in the country, which connects Brazil from north to south.

### 2.1. Formation of heavy mineral deposits

Ancient systems of paleochannels cutting the current emerging continental shelf (17 thousand years ago) are found in Rio Grande do Sul on its coast [24]. As sea levels rose during the post-glacial transgression (5,000 years ago), the channels were drowned and the trapped sediments were reworked, incorporating minerals into the coast [12, 25]. Upstream, these paleochannels are associated with the courses of the main rivers that drain the highlands to the north and the Precambrian basement in the center (southeast drainage basin) of the state, such as the Jacuí, Sinos, Taquarí, and Camaquã rivers [24]. The igneous and metamorphic rocks of the Riograndense South Shield are the likely source of the heavy mineral deposits found on the central coast [9, 24, 25].

**Fig 2:**
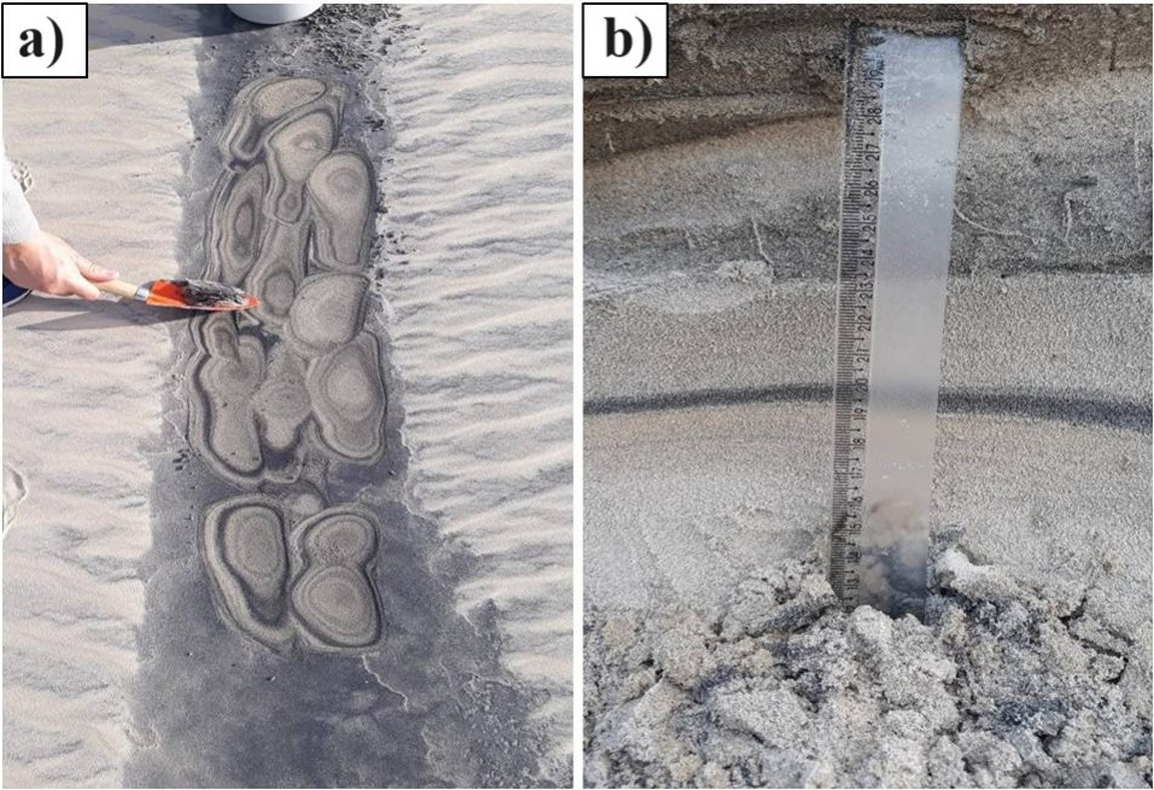
Retiro heavy mineral deposits, São José do Norte, Brazil.

The formation of the placer deposits is associated with long-term factors, such as the evolution of coastal barriers, and short-term factors, such as meteorological tides that erode foredunes and concentrate heavy minerals [12]. Wave action selects and concentrates the heavy minerals by hydraulic equivalence and the prevailing northeast winds transport the sand rich in heavy minerals inland in the form of transgressive dunes [25] (Fig 2A and 2b). The deposits extend below barrier IV (Holocene) and are anchored at barrier III (Pleistocene). The formation would have started in the last thousand years according to carbon-14 dating studies of the basal peat layer and continues to be formed to date [12].

### 2.2. Mining area–the Retiro project

The Retiro Project of Rio Grande Mineração Limited Liability Company (LLC). corresponds to the southern sector of the so-called South Atlantic Mining Complex. It is located in the rural area of SJN, where nearly 30% of the city’s population lives, and covers an area of 49 km^2^, being the only mining sector in the licensing process at the Brazilian Institute of Environment and Renewable Natural Resources (known as IBAMA). The other sectors (the central and the north) correspond to Capão/Estreito and Bojuru. The South Atlantic Mining Complex has a total area of 105 km^2^. The Retiro Project’s mining area extends over a strip having dimensions 1.5 km (width) and 30 km (length), from north of the urban area of São José do Norte in an easterly direction, until it crosses the BR-101, where it will follow parallel to the coastline, keeping 300 m away from the frontal dunes [11].

## 3. Data and methodology

### 3.1. Orbital and proximal data

Launched in 1999 through the collaboration of the space agencies of the United States (National Aeronautics and Space Administration–NASA) and Japan (Japan Aerospace Exploration Agency–JAXA), the ASTER sensor is on board the Terra multi-instrumental orbital platform, from the EOS (Earth Observing System) series. The ASTER sensor was developed with a focus on geological mapping, with its spectral bands positioned in wavelength ranges that allow differentiation of the spectral response of different mineral constituents. For this, ASTER has 14 bands, 5 bands in the thermal infrared TIR with a spatial resolution of 90 m, 3 bands in the visible and near-infrared (VNIR) with a resolution of 15 m, and 6 bands in the shortwave infrared (SWIR) with 30 m resolution [26]. ASTER’s TIR bands are positioned in the two atmospheric windows (between 8–9 μm and 10–12 μm) in which the influence of atmospheric constituents, such as ozone and water vapor, is lowest [27, 28], especially in coastal areas with higher humidity [29], as in the case of our study area, on the southern coast of Brazil.ASTER’s five TIR bands provide good spectral resolution for mapping rocks and minerals around the world, as many geological targets have their diagnostic spectral features in TIR [30–32].

Laboratory spectroscopy is a non-destructive mineral analysis technique that allows the comparison of hyperspectral data with multispectral data from orbital sensors [33, 34]. Minerals such as silicates have the Reststrahlen feature between 9 and 10 μm, which is the minimum emissivity value associated with the vibration of the Si-O molecular bond [35]. Ilmenite (FeTiO_3_) is an opaque mineral that does not present a spectral feature in VNIR-SWIR, with a straight and homogeneous spectrum. Oxides such as hematite, magnetite, and ilmenite, present spectral features above 10 μm [36]. The compilation of laboratory spectra provides spectral libraries that make the spectra of the main rock minerals available in digital format. It is a practical way of obtaining endmembers of interest, containing descriptive data regarding spectrum acquisition and purity of the analyzed sample [37, 38].

The key parameters for thermal remote sensing are surface emissivity (LSE) and surface temperature (LST), retrieved from surface thermal spectral radiance. Different algorithms were developed for this calculation [39, 40], based on the radiative transfer equation and Planck’s equation for blackbody radiation; the TES (Temperature Emissivity Separation) method [41] is the most used in the recovery of LSE for mineral substrates.

The NASA Land Processes Distributed Active Archive Center (LP DAAC) provides on-demand image surface emissivity (LSE) products. The level 2 processing product provides the 5 bands with LSE values recovered from the TES (Temperature Emissivity Separation) method [41] with ±0.015 accuracy [42, 43]. An uncertainty factor in emissivity retrieval is the contribution of scattered radiance emitted by the atmosphere within the sensor’s instantaneous field of view (IFOV) [44]. The atmospheric parameters for the correction are entered into the Moderate-resolution atmospheric Transmission (MODTRAN) radiative transfer mode [45], obtained from the Global Data Assimilation System

(GDAS) [46, 47]. Validation work on the ASTER surface emissivity product that used LSE measurements obtained in situ from pseudo-invariant targets presented results with high accuracy compared to the LSE values of the ASTER products for the same areas [48–51].

### 3.2. Methods

**Fig 3:**
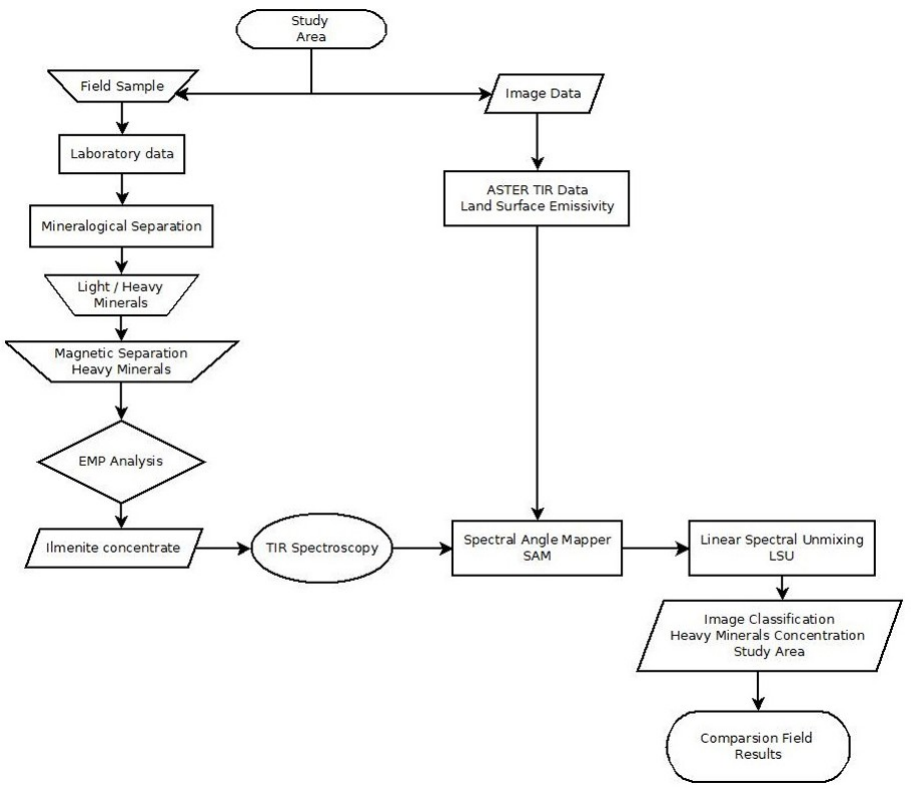
Flowchart of the methodology applied in this study.

In our study, we used the spectral signature of heavy minerals (from 8 to 14 μm) obtained with a Nicolet 6700 Thermo Scientific FT-IR spectrometer as an endmember reference to the digital image classification algorithms (SAM and LSU) on ASTER surface emissivity product (LSE) image for the study area. The purity of the heavy mineral samples was previously assessed by scanning electron microscope and analysis of characteristic X-rays. A flowchart of the methodology is shown in Fig 3.

#### 3.2.1. Sediment sample processing

Sediment samples were collected from the dune field, where a higher concentration of heavy minerals was found. After conditioning, the material was taken to the sample separation laboratory at the Center for Studies in Petrology and Geochemistry (CPGq), Institute of Geosciences, Federal University of Rio Grande do Sul (UFRGS). Then, hydraulic separation was carried out between light and heavy minerals using a drum disc. The portion of heavy minerals retained in the disk was dried in an oven, and polished section mounts were prepared for analysis under a scanning electron microscope.

#### 3.2.2. Scanning electron microscopy and X-ray spectrometry

Scanning electron microscopy (SEM) studies were realized at *Laboratório de GeologiaIsotópica* (CPGq-IGEO-UFRGS), including backscattered electron (BSE) imaging and chemical characterization with an EDS. SEM and standardless semiquantitative studies using energy-dispersive X-ray spectrometry (EDS) were carried out in the polished thin sections [52]. The SEM is a JEOL JSM-6610LV, equipped with a Bruker XFLASH 5030 energy dispersive X-ray spectrometer. Analytical conditions were 15 kV, spot size 55, working distance (WD) 11.2 mm, and counting time 30 s for the EDS analyses.

#### 3.2.3. Spectroscopy measurements of heavy minerals

The sample of heavy minerals was measured using a Nicolet 6700 Thermo Scientific FT-IR spectrometer, number of scans = 64 and a resolution of 0.2 μm at the Multiuser Thermal Analysis Laboratory (LAMAT) of the UFRGS under conditions of controlled temperature (20°C). The equipment was cooled with liquid nitrogen until the internal noise was stabilized. To calibrate the sensor and remove the background spectrum, a gold plate was used as a reference. The heavy minerals were mixed with potassium bromide (KBr) and diffuse reflectance measurements were taken of the sample in the range of 2.5 to 20 μm [53]. We only use values between 8 μm and 14 μm, common to geological remote sensing. The values measured in diffuse reflectance were converted to emissivity [33, 54, 55], according to Kirchoff’s Law (Eq 1) given below:

ε=1−R (1)

where, ɛ = emissivity and R = reflectance

Then, the spectral signature of the heavy mineral sample was resampled to the central value of the bandwidth compatible with the TIR ASTER data

#### 3.2.4. ASTER digital image processing

ASTER land surface emissivity (LSE) data for the study area was acquired on demand through the Land Process Distributed Active Archive Center (LP-DAAC). The LSE product has level 2 processing (L2) for the TIR bands of the ASTER sensor. Pixel-by-pixel LSE is estimated using the TES (Temperature Emissivity Separation) algorithm [41] for the five TIR bands with a spatial resolution of 90 meters (Table 1). The accuracy of the LSE product depends on the accuracy of the surface thermal radiance product, which uses the MODTRAN® radiative transfer model to correct for atmospheric effects [56]. Image processing was carried out using the Environment for Visualizing Images (ENVI) version 5.0 and ArcGIS 10.8 at the State Research Center for Remote Sensing and Meteorology (CEPSRM), Federal University of Rio Grande do Sul (UFRGS).

**Table 1:** Spectral information of ASTER bands used.

ASTER TIR	Band 10	Band 11	Band 12	Band 13	Band 14
**Spectralrange (μm)**	8.125–8.475	8.475–8.825	8.925–9.275	10.25–10.95	10.95–11.65
**Spacial Resolution**	90 meters				

As image classification algorithms, we applied SAM and LSU using the hyperspectral signature of the heavy mineral sample resampled for the response function of the ASTER TIR bands as input data (reference endmember) (Table 1). From the SAM, we identified the pixels containing the target of interest (heavy minerals), which were filtered to apply LSU to these pixels to estimate the abundance of the target within the pixel.

#### 3.2.5. The SAM algorithm

SAM, which is widely used in geological remote sensing studies [57–59], is based on the spectral similarity between reference endmember vs. pixel to classify the image pixel by pixel [60]. Spectral similarity is calculated through the average angle formed between the pair of spectra, with the spectra treated as vectors in an n-dimensional space (n = number of bands) [17], according to Eq 2.


$$\Theta =cos^{-1}\left(\frac{\sum_{i=1}^{n}m_{i}r_{i}}{{\left(\sum_{i=1}^{n}m_{i}^{2}\right)}^{\frac{1}{2}}{\left(\sum_{i=1}^{n}r_{i}^{2}\right)}^{\frac{1}{2}}}\right)$$ (2)

Where cos^-1^ is the angle between the two spectra.

SAM classification generates two output images, rule, and binary. The digital number (DN) values of the SAM rule image, in grayscale, express the value of the average angle between the pair of spectra. These values are weighted between 0 and 1, and the closer to zero the greater the similarity, represented by brighter pixels. In the binary SAM image, values that are within the established angular threshold will appear in white, while values outside the threshold will appear in black [19].

#### 3.2.6. The LSU algorithm

The LSU is an image classifier at the subpixel level based on spectral decomposition into smaller fractions, which allows for estimating the quantity of each target within the pixel [61]. Mathematically, the spectral response (gray level) of each pixel per band is the linear combination of the spectral responses of each constituent endmember, according to Eq 3.


rk=sk1f1+sk2f2+sknfn+i (3)

Where *rk* is the spectral response of the pixel in the band *k*; *sk* is the spectral response of the endmember in the *k* band; *fk* is the endmember proportion; and *i* = indeterminate portion.

The number of linear equations is equal to the number of bands in the image. An ASTER multispectral image with 5 TIR bands has 5 equations per pixel, and n unknowns per equation (n = endmembers). Each equation will have mathematical restrictions: 1) Each fraction must have a value between 0 and 100%, and 2) The sum of the fractions and undetermined portions must be equal to 100% [62].

The LSU approach involves endmember spectrum acquisition and ratio estimation procedures. Spectral mixture models have multiple applications in the remote mapping of natural resources, which is proven to be a useful technique for converting spectral information into the abundance product of the target of interest [13, 16, 63].

#### 3.2.7. Validation with field data

To compare the quantification of the surface distribution of heavy minerals obtained by mapping with the ground truth, we used data obtained in a previous study in the same study area [64] that carried out 33 samples from equidistant points within 300 meters, in an area of 1 Km x 3 Km. The author analyzed the concentration of heavy minerals in the subsurface with cores 1 m deep in 10 cm aliquots, totaling 10 aliquots per core. Only the aliquots from the first 10 cm layer were used as a reference, comparable to the surface mapping by remote sensing.

## 4. Results and discussion

The focus of the present study was on quantifying the concentration of heavy minerals, with the study area located on the coastal plain in the town of Retiro, municipality of São José do Norte, in the state of Rio Grande do Sul, Brazil. The present study integrated proximal and orbital remote sensing data in the thermal infrared, applying two supervised digital image classification techniques, namely SAM and LSU using an ASTER LSE Scene and spectral signatures of an assemblage of heavy minerals, previously collected in the field. In addition, the sampling of heavy minerals from the study area and analysis using scanning electron microscopy and x-ray spectrometry indicated ilmenite as the main mineral that makes up the assemblage of heavy minerals in the study area.

### 4.1. Electron microscopy analysis

**Fig 4:**
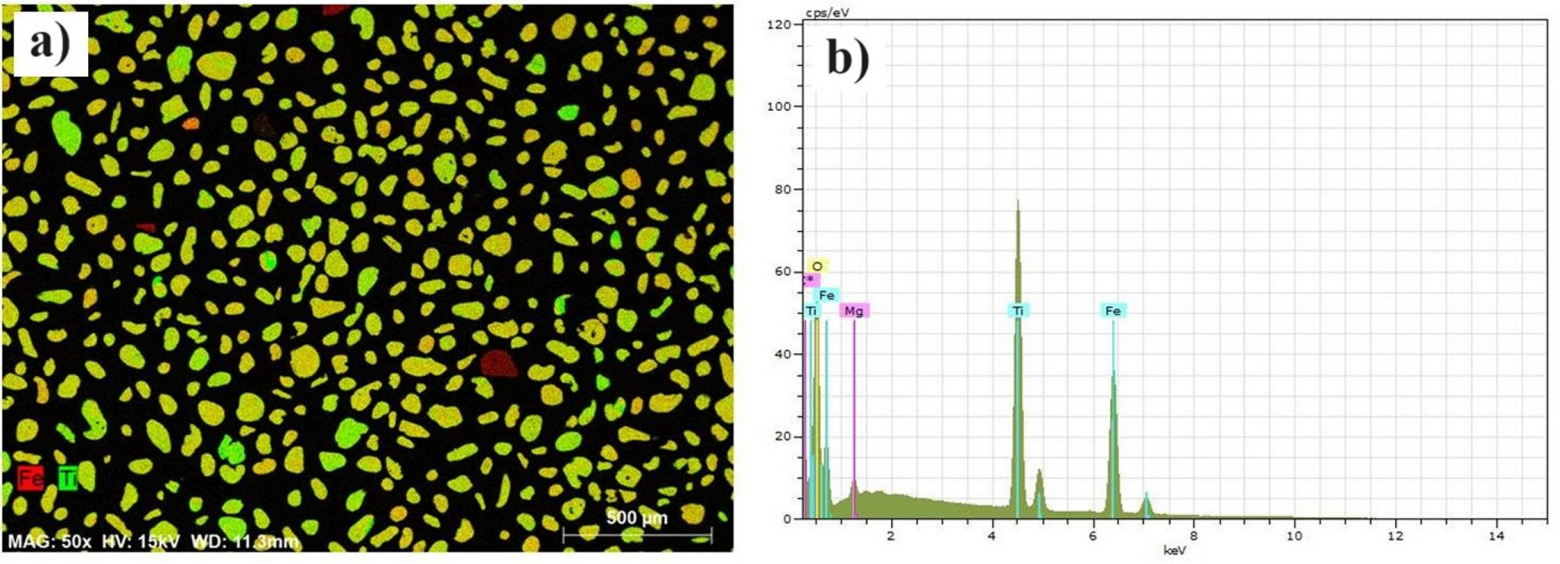
Electron microscopy analysis: a) characteristic X-ray map with EDS 1050 detector, and b) composition spectrum of an ilmenite grain.

In a stage before laboratory spectroscopy, the sample of heavy minerals collected in the field was analyzed by SEM to determine the composition and degree of purity of the sample, as highlighted by various authors [33, 35, 36, 55]. We used the Energy Dispersive Spectrometer (EDS)sensor to perform a qualitative analysis of the characteristic x-rays, which generated an elemental map of the sample (Fig 4A), and quantitative spot microanalysis of the content of the elements that make up the grains (Fig 4B).

The qualitative analysis of the characteristic X-ray maps shows the predominance of ilmenite (FeTiO_3_) in the image (Fig 4A) with the elements titanium (green) and iron (red) combined, classified according to their respective atomic masses based on backscattered electrons. Ilmenite has a theoretical composition of 53% TiO_2_ and 47% FeO and can undergo a process of alteration with iron leaching, increase in titanium content, and adsorption of impurities such as Al_2_O_3_ and SiO_2_ in the pores of the grains [65]. In quantitative microanalysis of the grains, we obtained a total of 66 spectra with the elemental composition of each of the 66 grains analyzed and most of the grains were identified as ilmenite (n = 41). In the spectrum shown in Fig 4B, it is possible to observe the Kα and Kβ peaks (at 4.5 keV) of titanium and iron (at 6.4 keV), and the presence of magnesium that enters the crystalline structure of ilmenite replacing the leached iron [65].

### 4.2. Spectral signature of heavy minerals in the laboratory

After analyzing the sample of heavy minerals under an electron microscope, we carried out spectroscopy measurements at TIR (8–14 μm) with a Nicolet 6700 FT-IR Thermo Scientific spectroradiometer. The diffuse reflectance values, collected in the laboratory test, were converted to emissivity, according to Kirchhoff’s Law. This is possible because the geometry of the laboratory measurement took place in hemispherical reflectance, for quantitative comparison with orbital imagery [33, 66].

**Fig 5:**
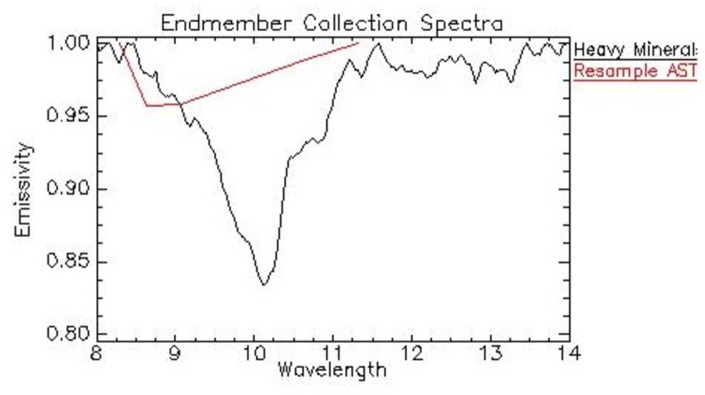
Spectral signature of the heavy mineral sample obtained with Nicolet FT-IR 6700 and resampled spectral signature for the ASTER TIR bands.

The spectral signature in the thermal spectrum range of the heavy mineral sample presents a maximum emissivity behavior (values close to 1) between 8 μm and 9 μm. From a wavelength of 9.3 μm, the emissivity values decrease until reaching a minimum peak at a wavelength of approximately 10.3 μm. This feature of the sample response spectrum is known as the Reststrahlen feature. At wavelengths starting from 10.5 μm, the emissivity values increase until reaching a maximum emissivity (values ~1), at a wavelength of approximately 14 μm (Fig 5).

Thus, with the spectral signature of heavy minerals, we resampled in terms of central bandwidth value for the response function of the TIR bands of the ASTER sensor, allowing the coincidence of the spectral intervals between the laboratory data and those from the ASTER sensor. Therefore, the spectral signature was compatible in wavelength coverage with the TIR multispectral bands of ASTER imagery.

In the resampled signature, the maximum emissivity value is observed in band 10, with a decrease between bands 10 and 11, and a subsequent gradual increase among bands 11, 12, and 13, respectively, until reaching the maximum emissivity value in band 14 (Fig 5). The absorption peak (Reststrahlen feature), observed in the spectral signature of heavy minerals, may be associated with the molecular vibration of the FeTiO_3_ bond (i.e., ilmenite), which corresponds to the main mineral component of the heavy mineral assemblage in the study area.

Ilmenite is a heavy opaque mineral, black in color, submetallic in luster, weakly magnetic, with a trigonal crystalline structure, with the elements iron and titanium alternating in layers of Fe^2+^ and Ti^4+^ ions linked to oxygen atoms [6]. Opaque minerals, such as magnetite and ilmenite, absorb a large part of the incident electromagnetic radiation, without presenting significant spectral features in the visible-near infrared (VNIR) and shortwave infrared (SWIR) wavelengths, presenting a restriction for remote sensing applications in these spectral ranges, due to the low signal-to-noise ratio in this wavelength range [67].

The shape and position of the absorption spectral bands, composing their spectral features (endmembers), are the diagnostic features of each mineral group [54]. These features derive from the selective absorption of photons, characterized by quantized energy values (i.e., associated with their frequency), necessary to excite the bond between molecules, according to the properties of the bond within the crystalline structure (e.g., bond strength, atomic mass, etc.) [35]. These vibrations can be stretching (i.e., symmetric or asymmetric), which changes the interatomic distance, or torsional, which changes the bond angle between atoms, altering the molecular geometry [68].

The Spectral Libraries available in digital repositories, such as ASTER JPL (Jet Propulsion Laboratory/NASA), USGS Spectral Library (United States Geological Survey), and JHU (Johns Hopkins University), among others, contain thousands of mineral spectra, representing a practical resource to geological mapping by remote sensing [37]. Even so, these libraries have limitations in terms of mineral coverage and the specific presentation of minerals from areas that do not correspond to the places that make up these collections.

Therefore, in the present study, we chose to create our spectral library based on samples of heavy minerals from the coast of Rio Grande do Sul. According to Beddel et al. [69], spectra obtained from samples collected from the study area better characterize the target of interest, which may undergo chemical changes specific to the location of occurrence. This is the case of the ilmenite alteration process that results from weathering, with iron leaching and an increase in the titanium content and other impurities in the grain [65, 70]. However, according to Lane and Bishop [54], these minor impurities are not capable of affecting the spectral expression of the dominant mineralogy in the TIR.

Iglesias et al. [71] studied the possible relationship between spectral signatures in the thermal range (TIR) and the geochemical compositions of volcanic rocks in the Paraná Basin, Brazil. The authors identified that the position of the spectral feature moved to longer wavelengths with the decrease in TiO_2_ content (i.e., ilmenite and titano-magnetite) and the increase in FeO. Christensen et al. [36] presented emissivity spectra of a series of oxide minerals, such as ilmenite, chromite, hematite, rutile, goethite, and magnetite, which show the variation in the shape and position of fundamental absorption features at wavelengths greater than 10 μm, associated with metal-oxygen stretching vibration. Ciazela et al. [72] identified prominent spectral features (between 20–40 μm) in oxides such as ilmenite when prospecting lunar deposits via remote sensing. These examples highlight the spectral specificities of endemic samples, reinforcing the contribution of collecting samples in the field to determine their spectral behavior, before mineral mapping via remote sensing.

Ytagesu et al. [73] analyzed the spectral behavior between the wavelength range of 2.5 and 14 μm of three pure clay minerals (i.e., montmorillonite, illite, and kaolinite) as well as the spectral behavior of known mixtures between them in the laboratory. From the different proportions of montmorillonite/kaolinite, montmorillonite/illite, and kaolinite/illite, the authors identified changes in the spectral behavior of the signatures, demonstrating the applicability of spectroscopy in the identification and quantification of clay minerals. According to the authors, the spectra of the separated minerals are characterized by absorption bands present between wavelengths of 2.5 and 3.7 μm, attributed to the hydroxyl group and H_2_O molecule, and by an absorption peak at 6.1 μm due to the bending vibration of the molecules of structural water. In montmorillonite/kaolinite mixtures, the absorption feature caused by the vibration of water molecules at 3.1 μm shifts to longer wavelengths as the kaolinite content increases, while the depth of the same feature increases as the montmorillonite content increases. The variation in the montmorillonite/kaolinite ratio also caused changes in the spectral behavior of the mixtures between 8 and 14 μm, better highlighted by the removal of the continuous spectrum (continuum removal). Mixtures of montmorillonite/illite and kaolinite/illite also showed spectral changes depending on the proportion of these minerals.

Laakso et al. [74] investigated the spectral behavior at TIR (between 8 and 12 μm) of economically important rare earth oxides using laboratory spectroscopy. The authors identified the Restrahlen feature in fluorocarbonate minerals (i.e., bastnasite and parysite) at approximately 11.5 μm, attributed to the vibration of the CO_3_^2-^ ion, with a slight shift of the feature to shorter wavelengths in the parysite curve. In the spectra of phosphate minerals, the monazite curve presents two absorption peaks at 9.5 and 9.75 μm, while in the xenotime signature, three spectral features were observed at 8.5, 9.5, and 9.75 μm, attributed to the molecular vibrations of the PO_4_^3-^ ion. In the silicate mineral samples (i.e., eudialyte, mosandrite, and zircon) the asymmetric stretching vibrations of the Si-O bond produced three spectral features (at 8.75, 9.5, and 10.5 μm) for the eudialyte sample, one feature (at 10 μm) for the mosandrite sample, and three features (at 8.75, 9.5, and 11 μm) for the zircon sample. However, the authors emphasize that none of the detected spectral features are exclusively diagnostic of rare earth minerals, but rather attributed to the fundamental vibrations of the C-O, P-O, and Si-O bonds, evidenced in other laboratory spectroscopy work for groups of carbonate minerals [75, 76], phosphates [77, 78], and silicates [35, 79]. This can confuse identification when using these features in remote sensing mapping.

Previous case studies reinforce the importance of determining the spectral signatures and characteristics of groups of minerals of interest, which aims to apply remote sensing mapping. This is due to the specificities of spectral signatures and their absorption features, making it vitally important to isolate and characterize spectral features that are exclusive to the mineral group that is sought to be identified, reducing the chances of classification misperception in mappings that are based on these data.

### 4.3. Heavy Minerals Occurrence in Retiro, the coastal plain of Rio Grande do Sul state

The integration of proximal (i.e., the spectral signature of heavy minerals) and orbital (i.e., ASTER LSE image) remote sensing data and the use of the SAM and LSU algorithms allowed us to map the occurrence and quantify the presence and distribution of heavy minerals over the surface of the Retiro dune field, in the coastal plain of Rio Grande do Sul. Similar approaches involving geological mapping using TIR data from the ASTER sensor, with endmember extraction and digital image processing, have been successfully applied in recent years [30], as well as in prospecting work for hydrothermal alteration zones associated with lead deposits [15], zinc deposits [13], and copper deposits [63] in Iran.

Pour et al. [16] mapped the occurrence of Listwanite rock in Antarctica as a product of hydrothermal alteration and an indicator of potential deposits of gold (Au), silver (Ag), mercury (Hg), and nickel (Ni), showing the potential of ASTER TIR data and image classification at sub-pixel level with LSU algorithm in mineral prospecting. From ASTER data, Gabr et al. [80] extracted the endmembers of mineralproducts of hydrothermal alteration and, using the SAM algorithm were able to detect zones of hydrothermal alteration with a high probability of gold mineralization in the Egyptian desert. Abubakar et al. [59] identified hydrothermal alteration clay minerals product (kaolinite, illite, muscovite, montmorillonite) indicators of geothermal systems in Nigeria, applying SAM and LSU on ASTER data.

**Fig 6:**
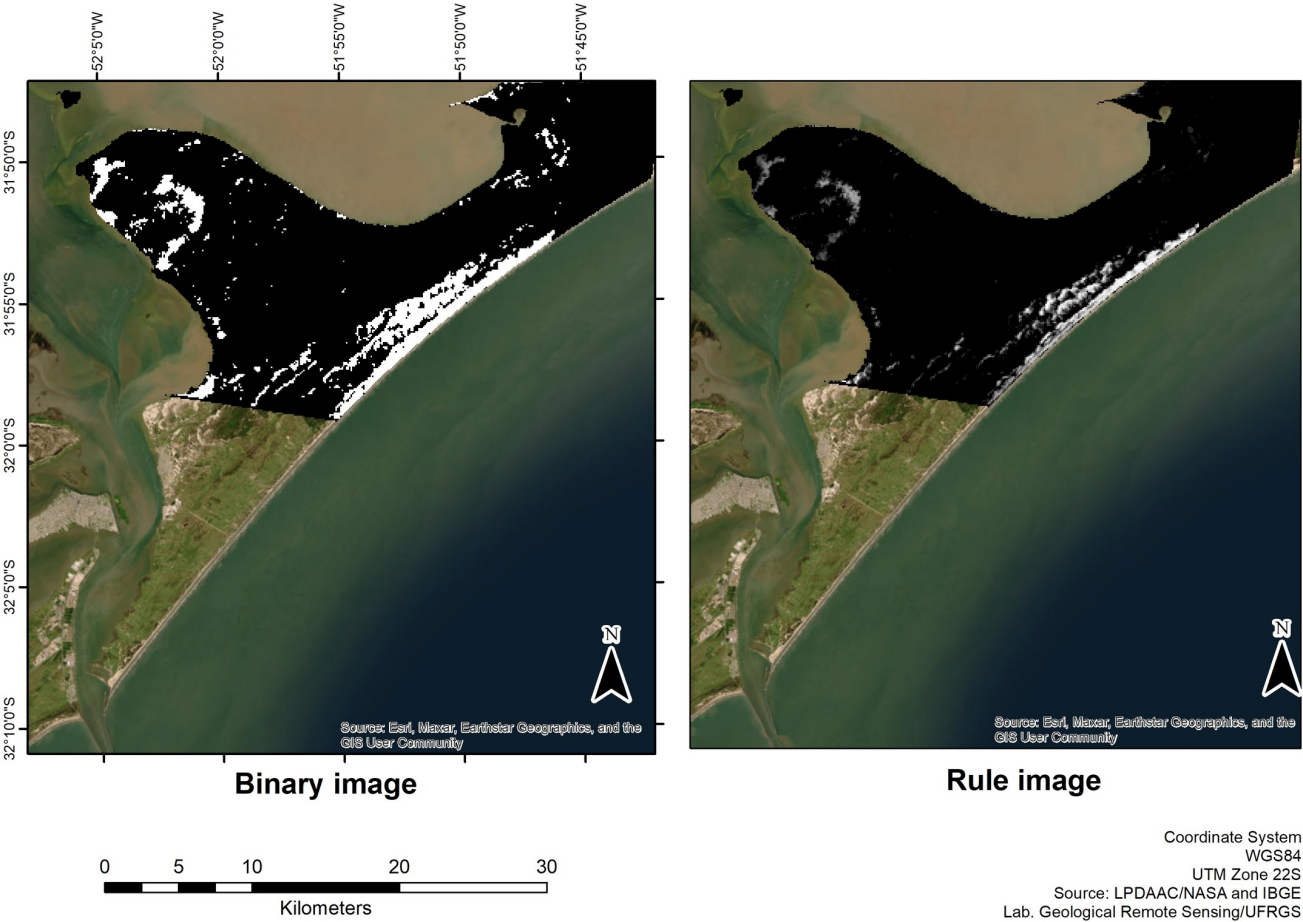
Rule and binary image (SAM) products showing Retiro heavy minerals deposit, Rio Grande do Sul, Brazil.

In the present study, the spectral signature of heavy minerals, measured in the laboratory, was resampled to match the spectral coverage of the wavelength values of the ASTER TIR bands (Fig 5), serving in sequence as reference endmembers for the applied SAM and LSU algorithms applied on ASTER LSE product covering our area of interest. Thus, from the SAM algorithm, two products were generated, a rule image, containing the spectral coincidence angles, and a binary image, containing the pixels within a pre-defined range of acceptable coincidence and representing the pixels of heavy mineral occurrence (Fig 6). The generated rule image, represented in gray tones, contains the digital number (DN) values, expressing the angular difference between the analyzed pair of spectra (i.e., reference endmember vs. pixel). DN values are weighted between zero and π/2, and the smaller the angular difference (closer to zero) the greater the spectral similarity. The binary image employs classification using a threshold angular value in which the pixel values within this threshold indicate the presence of the endmember (heavy minerals) in the pixel (in white) while pixels above the threshold indicate the absence of the endmember (in black) (Fig 6).

**Fig 7:**
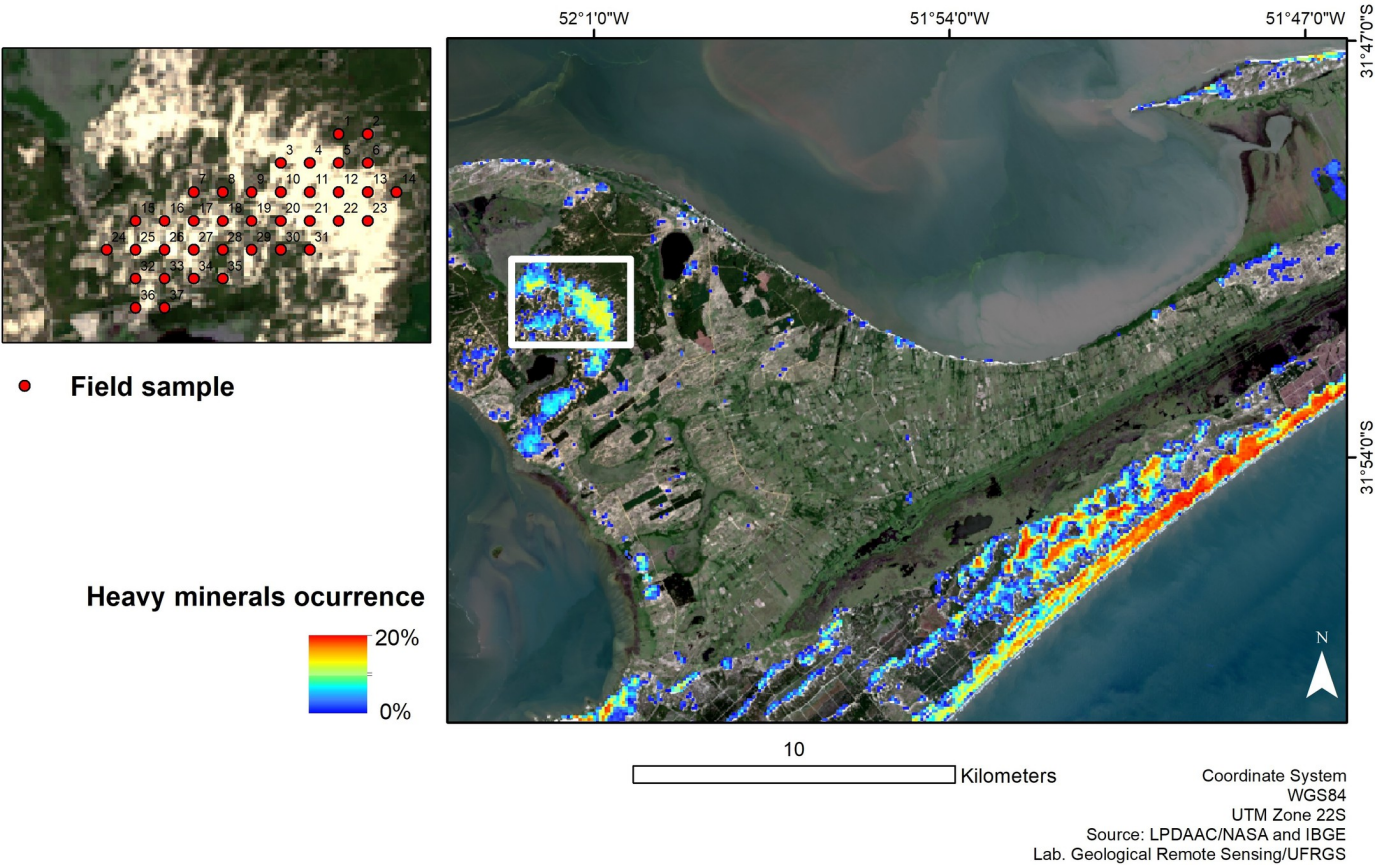
Linear Spectral Unmixing classification image showing the quantification of heavy minerals in a sector (Retiro location) of the coast of Rio Grande do Sul.

The pixels classified by SAM served as a mask for applying LSU, to estimate the concentration of heavy minerals at the sub-pixel level. Our results from the LSU classification indicate an average concentration of 8.8% of heavy minerals, with a maximum value of 20% and a minimum of 3.5% on the surface for the Retiro area, in the pixels where the sediment is exposed without vegetation cover (Table 2). In Fig 7, it is possible to observe a higher concentration of heavy minerals in the beach area and frontal dunes. The same pattern was observed by Munaro [81] who found an average of 4.6% of heavy minerals in the Bojuru coastal plain (50 km north of Retiro) with a concentration of up to 30% in the beach region. This is due to exposure to waves, which promote the selection and concentration of heavy minerals by hydraulic equivalence [12]. Barros et al. [82] identified near the Mostardas lighthouse (100 km north of Retiro) an average concentration of 15% of heavy minerals in the sediment. The study areas of Bojuru [81] and Farol de Mostardas [82] correspond to the same geological formation as Retiro, coastal barrier IV, of Holocene age [83].

**Table 2:** The concentration of heavy minerals in the study area based on the application of the Spectral Angle Mapper and Linear Spectral Unmixing algorithms.

Heavy Minerals Concentration–Retiro, São José do Norte, Brazil	
Mean	8.8%
Maximum	20%
Minimum	3.5%
Standard Deviation	4.6%

According to Dillenburg et al. [12], using carbon-14 dating of the basal peat layer, the formation of the Rio Grande do Sul placer deposit began in the last thousand years and is in continuous process today. The probable origin of these minerals is the igneous and metamorphic rocks of the Sul-Rio-Grandense Shield where paleo-drainage systems associated with the courses of the current Camaquã and Jacuí rivers cut through the coastal plain and current continental shelf [24, 84]. Sediments were reworked and deposited by short-term (e.g. waves, wind, tides) and long-term (e.g. sea level rise) coastal morphodynamic agents [85, 86].

To validate our remote sensing results, we used field data from Westphalen [64], 33 georeferenced point sediment samples, in which heavy mineral values from the field were superimposed on our SAM and LSU mapping. The concentration results of heavy minerals obtained in the field by Westphalen [64] indicate an average concentration of 1.9%, with a maximum value of 6.5% and a minimum of 0.3%. In comparison, our results indicated a higher average concentration per pixel for the same sampled area. Our LSU estimate presented an average result of 7.8% of heavy minerals, with pixels with a maximum value of 13.8% and a minimum value of 4.3% concentration (Table 3).

**Table 3:** Validation of heavy mineral quantification with field sampling.

Heavy Minerals Concentration	Field Data Westphalen [64]	Remote Sensing Data
Mean	1.9%	7.8%
Maximum	6.5%	13.8%
Minimum	0.3%	4.3%
Standard Deviation	1.6%	2.9%

Westphalen [64] identified that the concentration of heavy minerals increases with depth (up to 1 meter), due to the subsidence of these minerals with a higher specific weight compared to quartz. It reinforces our approach as a support for the geological mapping of coastal deposits of heavy minerals on the surface, as a way of making prospecting in large areas more agile, without replacing more detailed prospecting steps in the field.

According to Abrams and Yamaguchi [57], ASTER is the main orbital sensor for geological mapping due to its spectral resolution of 6 bands in SWIR (30 meters spatial resolution) and 5 bands in TIR (90 m spatial resolution), spectral regions where features occur diagnostics of mineral groups and rocks. Spectral mixing occurs regardless of the spatial resolution of the pixel, and the problem of pixel mixing is not rectified by improving the spatial resolution of the sensor [22]. Girouard et al. [87] analyzed the influence of the spatial resolution of sensors on geological mapping using endmembers extracted from images and applied to the SAM method. The authors compared the results obtained with VNIR-SWIR data from TM Landsat (30 m resolution) and QuickBird (1 m resolution) sensors for mapping rocks in Morocco. The results showed that despite the lower spatial resolution, the TM sensor produced maps with greater accuracy compared with the ground truth. According to the authors, this is because QuickBird does not have SWIR bands, and shows that spectral resolution is crucial for geological sensing.

Despite the widespread use of remote sensing data for geological prospecting and the importance of expanding the global supply chain for titanium ores [2, 5], studies that show the potential of remote sensing-based mapping of coastal mineral resources are still considered scarce. Among these, Chandrasekar et al. [67] used the Spectral Hourglass hyperspectral analysis tool implemented in ENVI^®^ software to map the distribution of heavy minerals off the coast of Tamil Nadu in India. The image processing steps include removing noise from the image (minimum noise fraction), identifying spectrally pure pixels (pixel purity index), and obtaining image endmembers. The image endmembers (i.e., zircon, ilmenite, garnet, and monazite) served as input to the SAM for classifying the ETM+ Landsat 7 image, and it was possible to map the pixels with a predominance of zircon, garnet, and monazite with high accuracy, but it was not possible to map ilmenite. Pixels classified as ilmenite were confused with quartz during field verification. The authors suggest that this is because the ilmenite signature is low reflectance and straight, with no significant spectral features in VNIR-SWIR.

Rejith et al. [88] mapped the heavy mineral deposits on the coast of Kerala. India, using VNIR-SWIR-TIR images from ASTER and Landsat. The authors used hyperspectral image analysis techniques to extract endmembers of interest from the data, which together with the SAM classifier, was able to find a high concentration of heavy minerals (>50%), with a predominance of ilmenite. Gazi et al. [14] mapped the occurrence of heavy minerals such as zircon, ilmenite, rutile, magnetite, and garnet using OLI Landsat 8 data. The authors also highlight geological mapping using an orbital sensor as an initial method for prospecting heavy minerals due to the special coarse resolution of the sensor. Ekanayake et al. [89] used hyperspectral data from the Hyperion sensor, laboratory spectral measurements, and data dimensionality reduction techniques to map the distribution of ilmenite off the coast of Sri Lanka with high accuracy.

Fabris [90] used remote sensing data to map coastal morphological features, and sandy ridges (dunes and paelodunes) in the Murray Basin, South Australia. The ASTER TIR bands were able to distinguish the type of vegetation of the ridges and valleys and thus map the dunes of Quaternary age, while the night-time TIR data (Landsat7 ETM+) were effective in detecting Tertiary paleodunes. The identification of these coastal features was important as it indicated the occurrence of heavy mineral deposits in the region. Dhinesh et al. [4] used Landsat 7 ETM+ data to map coastal drainage paleochannels as potential sites of heavy mineral deposits in Tamil Nadu, Southern India. Using digital processing techniques (pansharpening and PCA) to increase the spatial resolution of the image, and enhancement techniques (Gaussian stretching and Hue saturation value) the authors were able to visualize the pixels in white tones and the tonal variation towards the current course of rivers as the main key in the visual interpretation and identification of paleochannels. Excavations and field sampling confirmed the occurrence of paleochannels with concentrations of up to 15% of heavy minerals (predominantly ilmenite). The works of Fabris [90] and Dhinesh et al. [4] highlight TIR remote sensing as an economically viable and fast alternative method for mapping coastal resources.

The production of TiO_2_ and titanium metal from minerals, especially ilmenite, has grown considerably in recent decades. The magnetic properties of titanium increase the energy efficiency of reactors powered by oxygen combustion [7]. In addition, demand for titanium is growing to produce ceramics, anti-corrosive coatings, battery electrodes, medical and water treatment applications, and cosmetics [2] with an expected future increase in the prices of these commodities [5, 8]. In countries like Brazil, where exploration of heavy minerals is very low compared to potential, mapping new deposits represents an opportunity for economic growth [9, 10]. Remote sensing is a consolidated tool useful in aiding geological prospecting. However, heavy opaque minerals such as ilmenite and magnetite do not have significant spectral features in VNIR and SWIR regions, making their mapping by remote sensing difficult [67]. With this, the present study sought to innovate by using the potential of integrated TIR remote sensing techniques for the superficial quantification of the distribution of coastal heavy minerals, contributing to the few studies available in the literature on the identification of coastal mineral resources by remote sensing.

## 5. Conclusions

The present study quantified the distribution of heavy minerals of economic interest on the surface of the quaternary dune field in São José do Norte coastal plain in the state of Rio Grande do Sul, Brazil. The estimated heavy mineral concentration values reached up to 20% of the pixel. The highest concentrations occur close to the beach and frontal dunes, while in the internal dune field, the values are close to 10%. Using TIR data (8 μm to 14 μm) and digital image processing techniques, we innovate and show the potential of integrating proximal and orbital remote sensing data in this wavelength range for mapping coastal heavy mineral deposits.

Laboratory spectroscopy results using the Nicolet FT-IR 6700 showed a diagnostic spectral feature for a sample of heavy minerals, probably associated with the vibration of the ilmenite molecule (FeTiO_3_), the main heavy mineral species in the study area (>50%) verified by scanning electron microscopy analysis. The ASTER LSE image represents a simple and accurate method of obtaining surface emissivity values. The SAM algorithm was able to identify the pixels that contained spectral signatures of heavy minerals, while the LSU algorithm was able to estimate the proportion of the reference endmember in these pixels. Validation of classification results using previously collected local sampling points indicated high correspondence between heavy mineral concentration values in the field and pixels.

The present study paves the way for new research to be carried out with a similar approach in supporting geological prospecting for new heavy mineral deposits in coastal areas around the world. The global importance of exploring mineral resources rich in titanium, with demand and prices for these commodities anticipated to increase in the coming years. As perspectives for future work, we suggest expanding laboratory spectral signatures of other species of heavy minerals such as titano-magnetite, rutile, and zircon found in the study area, in addition to implementing approaches to applying artificial intelligence with machine learning to incorporate data of geological and physiographic interpretation that increase the mapping accuracy.
